# Comparative Discriminative Performance of Cast, Gap, and Three-Point Indices in Predicting Malunion in Elderly Patients with Conservatively Treated Distal Radius Fractures

**DOI:** 10.3390/medicina62040700

**Published:** 2026-04-06

**Authors:** Mehmet Maden, Mehmet Yiğit Gökmen, Tayfun Bacaksız, Cemal Kazımoğlu

**Affiliations:** 1 Department of Orthopaedics and Traumatology, Izmir Atatürk Training and Research Hospital, Izmir 35360, Türkiye; 2 Department of Orthopaedics and Traumatology, Faculty of Medicine, Canakkale Onsekiz Mart University, Canakkale 17100, Türkiye; mehmet_yigit_gokmen@hotmail.com; 3 Department of Orthopaedics and Traumatology, Faculty of Medicine, Izmir Katip Celebi University, Izmir 35620, Türkiye; tayfun_bacaksiz@hotmail.com (T.B.); ckazimoglu2000@yahoo.com (C.K.)

**Keywords:** distal radius fractures, conservative, elderly, malunion, orthopedic casts, radiographic measurements, predictive value of tests

## Abstract

*Background and Objectives*: Distal radius fractures (DRFs) are among the most common upper-extremity injuries in the elderly, with malunion leading to long-term pain and disability. This study aimed to compare the discriminative performance of the Cast Index (CI), Gap Index (GI), and Three-Point Index (TPI) for predicting malunion during follow-up after closed reduction and casting in elderly patients with dorsally displaced DRFs. *Materials and Methods*: This study retrospectively analyzed 274 patients aged ≥65 years (mean 73.6 ± 6.5 years) with dorsally displaced Arbeitsgemeinschaft für Osteosynthesefragen/Orthopaedic Trauma Association (AO/OTA) 2R3A fractures treated conservatively between 2018 and 2023. Standard posteroanterior and lateral radiographs were evaluated immediately post-reduction, at 7–10 days, and at 4 weeks. Cast, Gap, and Three-Point Indices were measured independently by two observers, and the mean values were analyzed. Receiver operating characteristic (ROC) analysis was used to evaluate discriminative performance for the detection of malunion. Subgroup analyses were performed based on fracture stability according to La Fontaine criteria. *Results*: At the final follow-up, 136 fractures (49.6%) maintained acceptable alignment, while 138 fractures (50.4%) experienced malunion. There was no significant difference in radiographic parameters between groups immediately post-reduction or at 7–10 days. The Gap and Three-Point Indices were significantly higher in the malunion group at 7–10 days and at 4 weeks (*p* < 0.001), but the Cast Index showed no significant between-group difference. At 7–10 days, the Gap Index [Area Under the Curve (AUC) = 0.641; cut-off = 0.33] and the Three-Point Index (AUC = 0.640; cut-off = 1.51) demonstrated modest discriminative ability. In stable fractures, both indices were statistically significantly higher in the malunion group, whereas in unstable fractures, only the Three-Point Index was statistically significantly higher in the malunion group (*p* < 0.001). *Conclusions*: The Gap and Three-Point Indices showed greater discriminative ability than the Cast Index for malunion after conservative management of DRFs in elderly patients. Thresholds of GI ≥ 0.33 and TPI ≥ 1.51 at 7–10 days may serve as practical quantitative indicators to identify patients who may warrant closer follow-up and possible cast reassessment in conjunction with standard radiographic assessment and clinical judgment.

## 1. Introduction

Distal radius fractures (DRFs) are among the most common upper-extremity fractures in elderly individuals, particularly in women [1]. With the ongoing demographic shift toward an aging population, the incidence of these fractures continues to rise. Malunion following DRFs has been shown to increase long-term pain, stiffness, and functional limitation, thereby posing a substantial public health burden in older adults [2,3]. While surgical fixation is often considered for younger or high-demand patients, the optimal management strategy for elderly patients remains controversial. Some meta-analyses suggest statistically better functional outcomes with volar plating in older patients, although not always reaching clinically meaningful thresholds [4,5,6].

Recent evidence suggests that nonoperative treatment via closed reduction and casting can yield acceptable functional outcomes in many elderly patients. In some randomized trials and meta-analyses, these outcomes are not significantly different from those achieved in surgically treated cohorts [7,8]. Accordingly, careful optimization of casting technique and close follow-up are essential to maintain reduction stability, as redisplacement remains a common and clinically relevant complication influenced by fracture characteristics, bone quality, and cast molding [9,10]. Current evidence-based recommendations for adult distal radius fractures emphasize individualized decision-making, underscoring the importance of early identification of patients at higher risk of redisplacement during conservative management [11].

Various radiographic indices, such as the cast index, gap index, and three-point index, were originally developed to assess cast quality and its relationship with loss of reduction in pediatric forearm fractures [12,13,14]. Among these, the cast index quantifies cast conformity, the gap index reflects soft-tissue–cast interface uniformity, and the three-point index evaluates the mechanical principles of three-point fixation. Although these indices are routinely used in pediatric populations, their clinical relevance and discriminative performance in elderly patients with osteoporotic bone remain unexplored. Evaluating the applicability of these indices in elderly patients may clarify whether they provide objective radiographic parameters to support early risk stratification during conservative management.

The present study aimed to evaluate the relationship between the cast, gap, and three-point indices and maintenance of reduction during conservative treatment of dorsally displaced distal radius fractures in elderly patients and to determine the extent to which these indices are associated with malunion and their discriminative performance for identifying malunion during follow-up.

## 2. Materials and Methods

### 2.1. Ethical Approval

All methods were carried out in accordance with relevant guidelines and regulations. This study was performed in line with the principles of the Declaration of Helsinki. Ethics approval was obtained by the Izmir Katip Celebi University Atatürk Training and Research Hospital Non-Interventional Clinical Studies Institutional Review Board (No:0055, Date: 15 February 2024).

### 2.2. Study Design and Population

This retrospective, single-center observational cohort study was conducted at Izmir Katip Celebi University Atatürk Training and Research Hospital (Türkiye) using hospital electronic medical records and the radiographic archive. Medical records and radiographs of patients aged 65 years or older who sustained dorsally displaced AO/OTA type 2R3A fractures between January 2018 and December 2023 were reviewed.

Patients were included if they were treated nonoperatively with closed reduction and short-arm plaster casting and if adequate radiographs were available at four time points: pre-reduction, immediately post-reduction, 7–10 days after reduction, and at 4 weeks post-injury.

**Figure 1:**
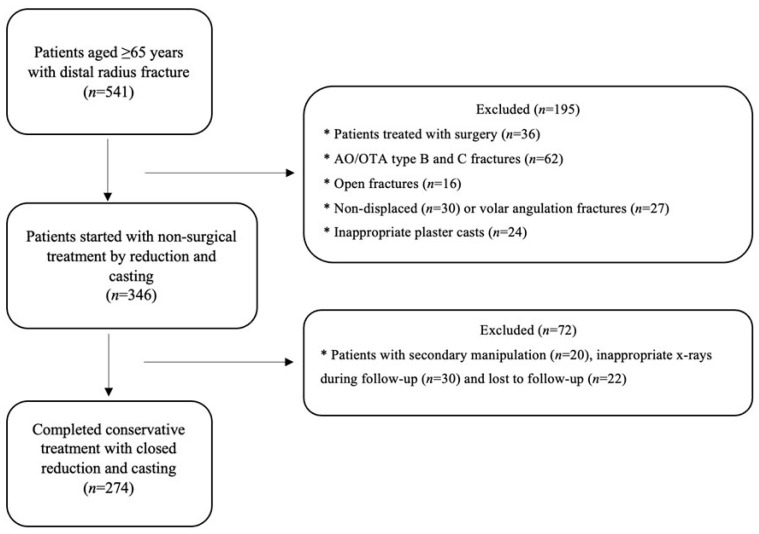
Flow diagram of patient inclusion and exclusion throughout the study.

Patients with volar angulation fractures, open injuries, AO/OTA type B or C fractures, nondisplaced fractures, secondary manipulation, inappropriate radiographs, or those who underwent surgery or were lost to follow-up were excluded (Figure 1).

### 2.3. Casting Technique and Follow-Up

Closed reduction was performed by orthopedic surgeons or senior residents under conscious sedation using manual longitudinal traction. A short-arm plaster cast was then applied with the wrist positioned at approximately 15° of palmar flexion and 10° of ulnar deviation, similar to the previous study and guidelines [11,15].

Patients were reassessed within 24 h to ensure proper cast tightness and were subsequently reviewed at 7–10 days and 4 weeks post-reduction with standard radiographs. Immobilization was maintained for at least 4 weeks, after which the cast was removed upon confirmation of radiological union. All patients were prescribed functional rehabilitation exercises thereafter.

### 2.4. Radiological Evaluation and Index Calculation

Radiological assessments were performed independently by an experienced radiologist and a trauma surgeon. Discrepancies were resolved by consensus. Standard anterior–posterior (PA) and lateral radiographs were used for measurements.

Fractures were classified according to the AO/OTA 2018 system [16]. The following parameters were recorded at each time point: dorsal tilt, radial inclination, ulnar variance, and the three radiographic indices (cast, gap, and three-point).

The indices were calculated as described in previous studies: The Cast Index (CI), originally described by Chess et al., represents the ratio of the internal diameters of the cast on lateral and posteroanterior radiographs at the fracture site [17]. This simple geometric relationship was intended to reflect how well the cast conforms to the limb in both planes.

The Gap Index (GI), later introduced by Malviya et al., quantifies the proportion of the cast–skin gap relative to the internal diameter of the cast in the sagittal and coronal planes, thereby offering a more detailed assessment of the interface between the cast and soft tissues [18].

The Three-Point Index (TPI), proposed by Alemdaroglu et al., expands upon these earlier measures by incorporating the proximal and distal contact regions in addition to the fracture level, reflecting the principle of three-point fixation that resists angular and translational displacement forces [19].

**Figure 2:**
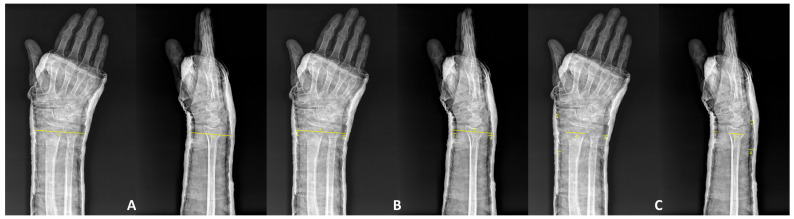
Schematic illustration of radiographic index measurements: (**A**) calculation of Cast Index, (**B**) measurement of Gap Index, and (**C**) Three-Point Index at three anatomical levels. These diagrams demonstrate the geometric relationship between the cast border and skin outline used for each index. All measurements were performed at standard PA and lateral wrist x-rays.

A schematic overview of these radiographic measurements is presented in Figure 2, illustrating (A) Cast Index, (B) Gap Index, and (C) Three-Point Index. Each index was measured immediately after reduction, at 7–10 days, and at 4 weeks by two independent observers. The mean of both readings was used for statistical analysis.

Interobserver reliability was assessed using the intraclass correlation coefficient (ICC) with a two-way random-effects model and absolute agreement. ICC values were interpreted as poor (<0.50), moderate (0.50–0.75), good (0.75–0.90), and excellent (>0.90).

### 2.5. Definition of Malunion and Fracture Stability

The acceptable alignment was defined based on recommendations from previous studies as follows: dorsal tilt > 100°, ulnar shortening > 3 mm, intraarticular step off > 2 mm, or radial inclination < 15° [20,21,22]. Dorsal tilt was recorded as the angle on the lateral radiograph, with values around 90° indicating neutral alignment; higher values indicate increasing dorsal tilt. Fractures without acceptable alignment were identified as malunions after cast removal, according to these criteria. All patients were divided into two groups: acceptable alignment and malunion, regardless of fracture stability, at the final radiological evaluation. Fracture stability was determined according to the La Fontaine criteria, which consider factors such as dorsal comminution, intra-articular involvement, ulnar styloid fracture, and patient age [23]. Patients were categorized into stable or unstable subgroups for secondary analysis.

### 2.6. Statistical Analysis

All statistical analyses were performed using SPSS version 22.0 (IBM Corp., Armonk, NY, USA). The normality of continuous variables was assessed using the Kolmogorov–Smirnov test. Normally distributed continuous variables were compared between groups using the Student *t*-test and are presented as mean ± standard deviation (SD). Because the study was retrospective, the sample size was determined by the number of eligible patients available during the study period. Therefore, the statistical power analysis was performed post hoc to evaluate the adequacy of the final sample size. Considering the gap index in the literature, it was calculated that a sample size of 274 patients (acceptable alignment/malunion distribution ratio 136/138) at an alpha level of 0.05 with an effect size of 0.8 would provide 99% power to detect a significant difference using the G*Power 3.1.9.7 program (Kiel, Germany) [18]. The primary analytical approach focused on between-group comparisons at each predefined follow-up time point to determine whether cast-related indices differed between fractures that maintained acceptable alignment and those that developed malunion. Formal within-group longitudinal comparisons across time points were not performed, as the main objective of the study was to assess between-group differences at clinically relevant follow-up assessments. To reduce the risk of Type I errors arising from the comparison of the three cast-related indices (Cast, Gap, and Three-Point) when patients were divided into subgroups based on stable versus unstable fractures at each follow-up time point, a Bonferroni-correction significance threshold of *p* < 0.017 (0.05/3) was applied. Receiver operating characteristic (ROC) analysis was performed to evaluate the discriminative performance of the indices for detecting malunion. For each ROC curve, the area under the curve (AUC) with its 95% confidence interval (CI), the optimal cut-off value based on the Youden index, and the sensitivities, specificities, positive predictive values, and negative predictive values were calculated. Except where the Bonferroni adjustment was applied, a *p*-value < 0.05 was considered statistically significant.

## 3. Results

### 3.1. Patient Characteristics

A total of 274 elderly patients (mean age: 73.6 ± 6.5 years; range, 65–94) with dorsally displaced distal radius fractures were included in the study. The cohort comprised 218 women (79.6%) and 56 men (20.4%). According to the AO/OTA 2018 classification, 78 fractures (28.5%) were type 2R3A2 and 196 (71.5%) were type 2R3A3. Based on the La Fontaine criteria, 132 fractures (48.2%) were classified as stable and 142 (51.8%) as unstable. The mean clinical follow-up period was 37 ± 7.2 months (range, 12–47). Baseline demographic and fracture characteristics are summarized in Table 1.

### 3.2. Comparison of Radiographic Parameters

Changes in dorsal tilt, radial inclination, and ulnar variance across follow-up intervals are presented in Table 2. No significant differences in radiographic parameters were observed between patients with acceptable alignment and those who developed malunion at either the post-reduction assessment or the 7–10-day follow-up (*p* > 0.05). By the final follow-up, significant differences emerged in all three parameters: mean dorsal tilt (91.7° ± 4.1° vs. 103.6° ± 7.5°, *p* < 0.001), radial inclination (22.0° ± 3.1° vs. 18.7° ± 3.5°, *p* < 0.001), and ulnar variance (0.9 ± 1.3 mm vs. 2.0 ± 1.8 mm, *p* < 0.001), confirming radiographic loss of reduction in the malunion group.

### 3.3. Comparison of Radiographic Indices

Interobserver reliability was consistently good to excellent across all time points. ICC values (two-way random-effects, absolute agreement) ranged from 0.82 to 0.91 for CI, 0.84 to 0.93 for GI, and 0.88 to 0.95 for TPI.

As shown in Table 2, no significant between-group differences were detected immediately after reduction for any of the evaluated indices. At 7–10 days, both the Gap Index and the Three-Point Index were significantly higher in the malunion group (Gap Index: 0.30 ± 0.08 vs. 0.26 ± 0.06, *p* < 0.001; Three-Point Index: 1.37 ± 0.33 vs. 1.19 ± 0.27, *p* < 0.001). At final follow-up, the between-group differences were more pronounced (Gap Index: 0.33 ± 0.08 vs. 0.28 ± 0.06, *p* < 0.001; Three-Point Index: 1.49 ± 0.28 vs. 1.28 ± 0.26, *p* < 0.001). In contrast, the Cast Index did not differ significantly between groups at any follow-up assessment.

These findings indicate that higher Gap and Three-Point Index values were observed in fractures that developed malunion and could be associated with malunion status at follow-up.

A comparison of the mean value of three indexes in patients with stable fracture patterns found no significant difference between the groups at post-reduction. The radiological evaluation of these patients at 7–10 days and 4 weeks post-injury revealed that the mean values of both the gap index and the three-point index were significantly higher in patients with malunion (*p* < 0.001) (Table 3).

The analysis of the mean values of three indexes in patients exhibiting unstable fracture patterns revealed no significant differences among the groups following post-reduction. The radiological assessment of these patients at 7–10 days and 4 weeks post-injury indicated that only the mean values of the three-point index were significantly elevated in patients with malunion (*p* < 0.001) (Table 3). All statistically significant findings remained significant after Bonferroni correction.

### 3.4. ROC Analysis in the Overall Cohort

**Figure 3:**
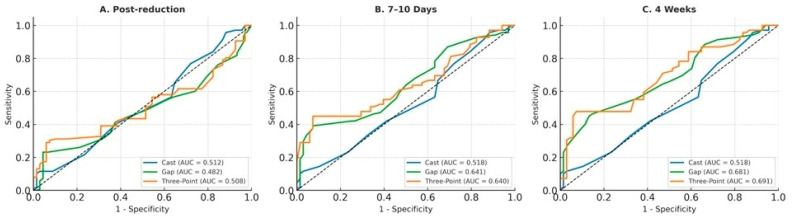
ROC curves for predicting malunion in the overall cohort at post-reduction (**A**), 7–10 days (**B**), and 4 weeks (**C**). Each panel demonstrates the discriminative performance of the Cast, Gap, and Three-Point indices in identifying fractures that developed malunion.

ROC curves for identifying radiographic malunion in the overall cohort at post-reduction, 7–10 days, and final follow-up are shown in Figure 3 and Table 4. The Gap Index and Three-Point Index demonstrated modest discriminative performance for malunion from the first follow-up onward. At 7–10 days, both indices showed statistically significant but limited discrimination, with high specificity and low sensitivity. At final follow-up, their discriminative performance improved slightly, whereas the Cast Index showed poor performance across all assessment time points. Overall, these findings suggest that the Gap Index and Three-Point Index were more informative than the Cast Index for distinguishing fractures that subsequently developed radiographic malunion during follow-up.

## 4. Discussion

This study evaluated whether plaster cast indices (Cast, Gap, Three-Point) could help discriminate malunion during follow-up in elderly patients undergoing non-surgical treatment for dorsally displaced distal radius fractures. Our findings revealed that while there was no significant difference in radiological parameters between groups at the post-reduction assessment or at 7–10 days, the Gap and Three-Point Indices showed modest but statistically significant discriminative ability for identifying malunion during follow-up from the first week onwards. Furthermore, the Three-Point Index appeared to be the most informative indicator among the evaluated indices, particularly in unstable fractures. Consequently, our results emphasize that for reduction maintenance in osteoporotic bone, not only the shape of the cast (Cast Index) but also the quality of cast conformity and pressure distribution may play an important role. In line with this, the proposed cut-offs demonstrated high specificity but relatively low sensitivity at 7–10 days, indicating that these indices are better interpreted as adjunctive risk stratification (warning) tools to identify a subgroup that may warrant closer monitoring for redisplacement rather than as screening tests to rule out malunion risk in the overall cohort.

Many prior studies of casting indices have focused on children and younger adults. Among adults, Alemdaroğlu et al. demonstrated the usefulness of the Three-Point Index, alongside the Cast and Gap Indices, in predicting the redisplacement of extra-articular distal radius fractures [19]. This gives methodological continuity to our choice of indices. In pediatric cohorts, indexed studies frequently report that poor molding, higher gap spaces, or suboptimal three-point support increase the risk of redisplacement. For example, Kong et al. found that in pediatric patients, a higher Three-Point Index and associated ulna fracture strongly correlated with redisplacement [24]. These pediatric observations affirm the principle that cast–skin gaps and pressure support are mechanistic contributors to stability, but they do not address skeletal or soft-tissue changes present in the elderly.

In geriatric populations, the relationship between radiographic alignment, malunion, and functional outcomes remains debated. Some series, including a registry-based review, suggest no clear superiority of surgical fixation over conservative treatment in terms of function at one year [2]. Among “superelderly” patients (≥80 years), malunion did not significantly affect grip strength, range of motion, pain, or daily living activities [25]. Additionally, Nelson et al. reported that among older adults, even active ones, malunion did not always translate into worse wrist disability [26]. These results suggest a degree of functional tolerance to anatomic deformity in low-demand elderly patients. On the other hand, Symonette et al. reported that malunion still has harmful effects in distal radius fractures in elderly patients [27]. From this perspective, attention should be paid to the development of malunion in the conservative treatment of DRF in elderly patients, and follow-ups should be prioritized.

Our temporal findings, including the lack of discriminatory separation immediately after reduction, the emergence of between-group differences by 7–10 days, and the stronger differentiation at four weeks, align with biomechanical expectations. Early after reduction, soft-tissue swelling and tightness of the cast may mask subtle mismatches; however, as edema resolves and the cast loosens, gaps and pressure imbalances become apparent. In recent work, Barvelink et al. quantified cast-molding quality in a mixed-age cohort [9]. They reported that poor molding, identified by thresholds of TPI, CI, and GI, correlated with the risk of redisplacement. Their observations reinforce our conclusion that casting quality contributes to reduced maintenance. However, they also show that casting is only one piece of the displacement puzzle.

Importantly, the discriminative performance of Gap and Three-Point Indices in our cohort is modest but clinically meaningful. Given that reduction maintenance is a multifactorial process, influenced by fracture morphology, bone quality, patient compliance, cast wear, and micro-movement, a single index is unlikely to be perfect. Yet, our results suggest that objective, reproducible measurements of cast–skin conformity may provide practical value as an adjunct to serial radiographic follow-up.

Clinically, the thresholds we identified (Gap Index > 0.33, Three-Point Index > 1.51 at 7–10 days) may guide decision-making. In elderly patients, particularly those who may not tolerate repeated reduction, these indices offer a quantitative supplement to routine assessment. Patients exceeding these thresholds may warrant consideration of earlier re-casting or closer radiographic monitoring in conjunction with clinical judgment and standard radiographic evaluation. Because the indices are derived from standard AP and lateral radiographs, they require no additional imaging or cost, making them feasible for use in routine practice.

Overall, our findings reinforce that cast–skin conformity and three-point pressure distribution, as quantified by the Gap and Three-Point Indices, were more frequently observed in fractures that subsequently developed malunion in the elderly. These indices should not supplant, but rather augment, fracture stability assessment. Given their limited sensitivity, they should not be used as stand-alone screening tools. Instead, when interpreted together with routine radiographic assessment, elevated values may serve as practical warning markers that support closer follow-up and timely clinical reassessment before malalignment becomes established.

The strengths of this study include a large and homogeneous cohort of elderly patients, good-to-excellent interobserver reliability, and ROC-based evaluation of cast indices at multiple time points. Nevertheless, several limitations should be acknowledged. First, the retrospective design inherently carries a risk of selection bias, although strict inclusion criteria and standardized measurement protocols were applied to reduce this effect. Second, functional and patient-reported outcomes were not assessed; therefore, the radiographic definition of malunion could not be directly correlated with long-term wrist function. This issue is particularly relevant in older adults, in whom radiographic and functional outcomes may not always correspond. Despite these limitations, the present study provides useful radiographic data that may support clinical follow-up and may serve as a basis for future prospective studies.

## 5. Conclusions

In elderly patients with dorsally displaced distal radius fractures treated conservatively, the Gap Index and Three-Point Index demonstrated greater discriminative performance than the Cast Index for predicting radiographic malunion during follow-up. However, given their limited sensitivity, these indices should not be used as stand-alone screening tools. Instead, threshold values such as GI ≥ 0.33 and TPI ≥ 1.51 at 7–10 days may serve as adjunctive, high-specificity indicators to support closer follow-up, cast reassessment, and clinical decision-making when interpreted together with standard radiographic evaluation.

## Figures and Tables

**Table 1:** Baseline demographics and fracture characteristics of the study population.

Variable	
Age [mean ± SD (min-max), years]	73.6 ± 6.5 (65–94)
Sex [male: female, *n* (%)]	56 (20.4): 218 (79.6)
AO Fracture classification	
2R3A2 *n* (%)	78 (28.5)
2R3A3 *n* (%)	196 (71.5)
Fracture stability [unstable: stable, *n* (%)]	142 (51.8): 132 (48.2)
Clinical follow-up period [mean ± SD (min-max), months]	37 ± 7.2 (12–47)

SD: Standard deviation, *n*: number.

**Table 2:** Comparison of radiographic indices between acceptable alignment and malunion groups.

Variable	Acceptable Alignment (*n* = 136) Mean ± SD	Malunion (*n* = 138) Mean ± SD	*p*-Value
Dorsal tilt (degrees)			
Pre-reduction	108.2 ± 5.5	107.9 ± 6.1	0.771 ^+^
Post-reduction	89.2 ± 3.6	89.5 ± 3.2	0.471 ^+^
After 7–10 days	91.3 ± 3.9	92.0 ± 3.5	0.116 ^+^
Final follow-up	91.7 ± 4.1	103.6 ± 7.5	<0.001 ^+^
Radial inclination (degrees)			
Pre-reduction	18.8 ± 4.4	19.1 ± 4.1	0.672 ^+^
Post-reduction	24.2 ± 2.9	24.2 ± 1.9	0.858 ^+^
After 7–10 days	22.9 ± 3.6	22.3 ± 1.9	0.117 ^+^
Final follow-up	22.0 ± 3.1	18.7 ± 3.5	<0.001 ^+^
Ulnar variance (mm)			
Pre-reduction	1.5 ± 2.1	1.6 ± 2.2	0.832 ^+^
Post-reduction	0.1 ± 1.4	0.3 ± 1.2	0.335 ^+^
After 7–10 days	0.7 ± 1.3	0.9 ± 1.2	0.079 ^+^
Final follow-up	0.9 ± 1.3	2.0 ± 1.8	<0.001 ^+^
Indexes (%)			
Post-reduction			
Cast index	0.83 ± 0.04	0.84 ± 0.04	0.195 ^+^
Gap index	0.23 ± 0.05	0.23 ± 0.07	0.986 ^+^
Three-point index	1.02 ± 0.28	1.05 ± 0.41	0.451 ^+^
After 7–10 days			
Cast index	0.83 ± 0.04	0.84 ± 0.04	0.148 ^+^
Gap index	0.26 ± 0.06	0.30 ± 0.08	<0.001 ^+^
Three-point index	1.19 ± 0.27	1.37 ± 0.33	<0.001 ^+^
Final follow-up			
Cast index	0.83 ± 0.04	0.84 ± 0.04	0.128 ^+^
Gap index	0.28 ± 0.06	0.33 ± 0.08	<0.001 ^+^
Three-point index	1.28 ± 0.26	1.49 ± 0.28	<0.001 ^+^

SD: Standard deviation, mm: millimeter, ^+^: Student *t*-test.

**Table 3:** Comparison of radiographic indices between acceptable alignment and malunion groups, based on stable or unstable fracture patterns.

Variable	Stable Fracture (*n* = 132)			Unstable Fracture (*n* = 142)		
	Acceptable (*n* = 56)	Malunion (*n* = 76)	*p*-value	Acceptable (*n* = 80)	Malunion (*n* = 62)	*p*-value
	mean ± SD	mean ± SD		mean ± SD	mean ± SD	
Indexes (%)						
Post-reduction						
Cast index	0.84 ± 0.03	0.83 ± 0.02	0.588 ^+^	0.83 ± 0.04	0.84 ± 0.05	0.094 ^+^
Gap index	0.23 ± 0.04	0.23 ± 0.07	0.840 ^+^	0.23 ± 0.06	0.22 ± 0.06	0.804 ^+^
Three-point index	1.08 ± 0.22	1.10 ± 0.45	0.684 ^+^	0.98 ± 0.31	1.01 ± 0.32	0.826 ^+^
After 7–10 days						
Cast index	0.84 ± 0.03	0.83 ± 0.02	0.722 ^+^	0.83 ± 0.04	0.84 ± 0.05	0.076 ^+^
Gap index	0.28 ± 0.05	0.33 ± 0.08	<0.001 ^+^	0.24 ± 0.06	0.26 ± 0.06	0.110 ^+^
Three-point index	1.24 ± 0.19	1.42 ± 0.34	<0.001 ^+^	1.16 ± 0.31	1.32 ± 0.32	<0.001 ^+^
Final follow-up						
Cast index	0.84 ± 0.03	0.83 ± 0.02	0.722 ^+^	0.83 ± 0.04	0.84 ± 0.05	0.061 ^+^
Gap index	0.29 ± 0.05	0.37 ± 0.08	<0.001 ^+^	0.27 ± 0.06	0.28 ± 0.06	0.117 ^+^
Three-point index	1.31 ± 0.19	1.54 ± 0.27	<0.001 ^+^	1.25 ± 0.30	1.43 ± 0.28	<0.001 ^+^

SD: Standard deviation, ^+^: Student *t*-test.

**Table 4:** Discriminative performance of cast-related indices for radiographic malunion at follow-up.

Time Point	Index	AUC (95% CI)	Cut-Off	*p* Value	Sensitivity (%)	Specificity (%)	PPV (%)	NPV (%)	Youden İndex
After 7–10 days	Gap index	0.641 (0.576–0.706)	0.33	<0.001	39.1	92.6	84.4	60.0	0.318
	Three-point index	0.640 (0.575–0.705)	1.51	<0.001	44.9	92.6	86.1	62.4	0.376
Final follow-up	Gap index	0.681 (0.619–0.744)	0.35	<0.001	44.9	86.8	77.5	60.8	0.317
	Three-point index	0.691 (0.629–0.753)	1.60	<0.001	47.8	92.6	86.8	63.6	0.405

AUC: area under the curve; CI: confidence interval; PPV: positive predictive value; NPV: negative predictive value. Optimal cut-off values were determined using the Youden index.

## Data Availability

The data presented in this study are available on reasonable request from the corresponding author. The data are not publicly available due to institutional privacy regulations.

## Abbreviations

The following abbreviations are used in this manuscript:

DRF	Distal radius fracture
CI	Cast Index
GI	Gap Index
TPI	Three-Point Index
ROC	Receiver Operating Characteristic
AUC	Area Under The Curve
AP	Anterior–posterior
mm	millimeter
*n*	number
SD	Standard Deviation
SPSS	Statistical Package for the Social Sciences
